# Improvement in and Validation of the Physical Model of an Intelligent Tire Considering the Wear

**DOI:** 10.3390/s25082490

**Published:** 2025-04-15

**Authors:** Guolin Wang, Xiangliang Li, Zhecheng Jing, Xin Wang, Yu Zhang

**Affiliations:** 1 School of Automotive and Traffic Engineering, Jiangsu University, Zhenjiang 212013, China; glwang@ujs.edu.cn (G.W.); 2212204009@stmail.ujs.edu.cn (X.L.); 2222204147@stmail.ujs.edu.cn (X.W.); zhangyu_ujs@163.com (Y.Z.); 2 Jingjiang College, Jiangsu University, Zhenjiang 212013, China

**Keywords:** intelligent tire, tire physical model, tire wear, flexible ring model, strain signal

## Abstract

The development of intelligent tire technology has attracted increasing attention from researchers to build different tire models to obtain the state parameters of the tire and to try to correlate these parameters with sensors. To address the challenge of characterizing the evolution of wear in traditional tire mechanics models, this study proposes a physical model that incorporates tire wear. The model is an improvement over the traditional flexible ring model, incorporating brush theory. By establishing the mechanical equilibrium equation of the tread unit, a tire dynamic equation incorporating wear state variables is constructed. The strain–displacement relationship is analyzed to determine the correlation between the strain field and the displacement field. The results show that the strain signals obtained from the physical model and the finite element model maintain a high degree of consistency, validating the reliability and effectiveness of the proposed model. In addition, the correlation between the tire wear and strain signal characteristics was successfully revealed by comparing the physical model and the finite element model. The proposed model provides a theoretical foundation for future research on intelligent tires, as well as a basis for related studies on tire wear, tire lifespan, and tire mechanical properties.

## 1. Introduction

The rapid development of intelligent driving technology promotes the innovation of vehicle perception systems. As the only carrier of the interaction between the vehicle and the road surface, the tire is not only the motion execution component but also a natural sensor that obtains road surface information. Compared to body sensors, tires directly carry the mechanical interaction of the vehicle–road interface. Physical signals such as tire strain and acceleration can more accurately reflect the tire–road contact state. This makes intelligent tire technology a key breakthrough to improve vehicle active safety and motion control performance. The early concept of intelligent tires originated from the tire pressure monitoring system (TPMS), which mainly realized basic safety functions such as tire burst warnings. With the advancement of sensing technology, modern intelligent tires have developed into “active” components that can sense external information [1], and their core goal is to obtain high-value parameters such as the tire six-component force, wear state, and road adhesion coefficient in real time. At present, primary intelligent tires based on tire temperature and tire pressure monitoring have been commercialized, and new intelligent tires with complex state perception capabilities still need more in-depth research.

In the field of intelligent tires, there are currently three major technical bottlenecks. One is sensing technology, the second is the signal transmission and power supply system, and the third is the estimation of state parameters. The first two aspects in the current development stage have effective solutions.

In terms of sensors, triaxial acceleration sensors [2,3,4,5], strain gauges [6,7,8,9,10], and PVDF piezoelectric films [11,12,13,14,15] have become mainstream sensing solutions due to their strong anti-interference and convenient installation advantages, while flexible sensors (microfluidic liquid metal sensors [16], laser-induced graphene sensors [17]) have also demonstrated better signal fidelity. Based on the consideration of signal stability and practicability, this paper chooses strain signal as the main research object. In terms of energy supply and signal transmission, both traditional wired transmission [10], slip ring transmission [18], and breakthrough self-powered technology [19,20,21] can fulfill the corresponding experimental requirements. However, the most critical technical challenge is the estimation of state parameters, and existing methods can be classified into two categories: data-driven machine learning and physical-model-driven. The former relies on machine learning technology to construct a prediction model through signal features. Scholars use algorithms such as Gaussian Process Regression (GPR) [22,23], a Support Vector Machine (SVM) [6,24,25], and Neural Networks [26,27,28], which show high accuracy in specific tasks such as the length of grounding imprints and the identification of slip states. However, there are limitations of high data acquisition costs and a weak model generalization ability. And a model-driven approach can precisely make up for the former disadvantages. Based on a physical model, the corresponding state observer can be constructed, and algorithms such as the Kalman filter [29,30,31,32] and wavelet transform [13,33] can be used to realize the estimation of mechanical parameters. Many scholars take the traditional two-dimensional flexible ring model as the theoretical basis and then combine various types of sensors for state estimation. D. Min [34] fused strain sensors with accelerometers based on a conventional flexible ring model, revealing the intrinsic connection between the different signals and estimating the pendant force with effective accuracy. X. Gao [35] measured the deformation of a high-speed rolling tire based on a flexible ring model combined with the digital image correlation (DIC) method and proved the deformation characteristics of a tire under high-speed rolling. D. Jeong [36] simplified the flexible ring model and defined a tire dimensionless number characterizing the tire properties based on the Buckingham PI theorem, which was verified using tests based on acceleration signals. Meanwhile, some scholars have also built a three-dimensional circular model to develop a lateral force estimator for intelligent tires, which combines the signal processing of acceleration sensors and the application of Kalman filtering to achieve the observation of the state of the lateral force [31]. It can be seen that the application of models is of great value in the study of intelligent tires. However, in actual use, tire characteristics are affected by wear and tear, and the above application of a model fails to achieve this. If the time-varying factors, such as tire wear, could be taken into account before estimating the state parameters, the estimation could be closer to the actual situation, thus improving the accuracy of the estimation. Therefore, this paper presents a new physical model that can take tire wear into account. At the same time, the model can be used to generate the required strain signals directly to produce a benchmark dataset. Thus, it can replace the finite element model or test. The model parameters are optimized in conjunction with the measured data to maintain the physical interpretability and enhance the adaptability to dynamic factors such as tire wear. This research paradigm can effectively reduce the cost of data acquisition and provide new ideas for the engineering application of intelligent tire technology.

The main research content of this paper is to take the tire flexible ring model as the basis, improve the model by combining the brush characteristics, and consider the tire wear in the form of the brush length in the model to explore the effect of wear on the strain signal of an intelligent tire. The improved theoretical model establishes a computable bridge between tire wear and measurable strain signals, which is essential for the design of strain-sensor-based intelligent tire systems. By deriving an explicit relationship between contact mechanics and strain distribution, the model enables sensor virtual data generation, which is a key step towards a low-cost and highly reliable embedded sensing solution. The structure of this paper is organized as follows: First, the basic idea of the flexible ring model and the improvement process of the model are introduced. Subsequently, the improved physical model is analyzed and solved to obtain the strain expression of the model. Then, the strain signals obtained from the model are verified by finite elements. Finally, the relationship between tire wear and strain signals is explored using the physical model.

## 2. Model Building

### 2.1. The Flexible Ring Model

**Figure 1:**
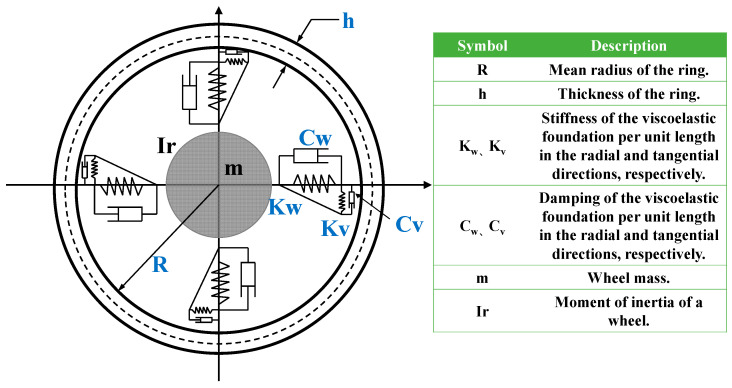
Schematic diagram of flexible ring model.

Since this study focuses on strain-based intelligent tire systems, a flexible ring model that can utilize strain is chosen. The flexible ring model considers the tire system as a two-dimensional ring model consisting of the tire tread, the tire sidewall, and the rim in which the tire tread is considered as a thin circular ring with homogeneous material properties, the tire sidewall and the compressed air are considered as a two-degree-of-freedom spring system distributed along the circumferential direction of the ring, and the rim is regarded as an axisymmetric rigid body with the mass and moment of inertia as shown in Figure 1.

**Figure 2:**
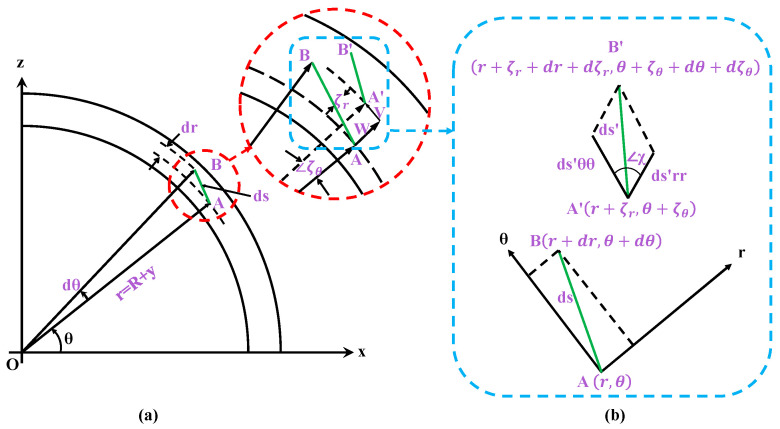
(**a**) Schematic diagram of the strain–displacement relationship for tire deformation; (**b**) change in the position of the point.

During tire movement, points on the tire are displaced, and rubber as an elastic material is bound to deform, so it is particularly important to obtain the relationship between displacement and strain on the tire. In Gong’s study, the strain–displacement relationship for the flexible ring model has been illustrated by a detailed derivation of equations [37]. For the flexible ring model, take the center of the ring as the origin, establish a polar coordinate system, and take two adjacent points A(r,θ) and B(r+dr,θ+dθ) on the ring (as shown in Figure 2) to study the strain–displacement relationship before and after the deformation of the ring model.

The length between two points ds can be approximated by a hypotenuse of a triangle:

(1) $${\left(ds\right)}^{2}\approx {\left(d\theta \right)}^{2}+{\left(dr\right)}^{2}$$

Assuming that after the deformation of the tire, point A undergoes a displacement change in W in the radial direction and V in the tangential direction, the polar diameter and the polar angle increase by $\zeta_{r},\zeta_{\theta }$, respectively, and the point B moves towards $B^{\prime }$ as shown in Figure 2a.

(2a) $$A\left(r,\theta \right)\to A^{\prime }\left(r+\zeta_{r},\theta +\zeta_{\theta }\right)$$

(2b) $$B\left(r+dr,\theta +d\theta \right)\;B^{\prime }\left(r+\zeta_{r}+dr+d\zeta_{r},\theta +\zeta_{\theta }+d\theta +d\zeta_{\theta }\right)$$

According to the geometric relationship, we can obtain the following:

(3a) $$\zeta_{\theta }\approx sin\zeta_{\theta }=\frac{V}{r+W}$$

(3b) $$\zeta_{r}=\sqrt{(r+W{)}^{2}+V^{2}}-r\approx W$$

Therefore, the length between the two points after deformation is as follows:

(4) $${\left(ds^{\prime }\right)}^{2}\approx {\left(r+\zeta_{r}\right)}^{2}{\left(d\theta +d\zeta_{\theta }\right)}^{2}+{\left(dr+d\zeta_{r}\right)}^{2}$$

where the differential for a minimal quantity can be expressed as:

(5a) $$d\zeta_{\theta }=\frac{\partial \zeta_{\theta }}{\partial \theta }d\theta +\frac{\partial \zeta_{\theta }}{\partial r}dr$$

(5b) $$d\zeta_{r}=\frac{\partial \zeta_{r}}{\partial \theta }d\theta +\frac{\partial \zeta_{r}}{\partial r}dr$$

Substituting Equation (5a,b) into Equation (4) yields a complex equation:

(6) $${\left(ds^{\prime }\right)}^{2}=G_{\theta \theta }{\left(d\theta \right)}^{2}+2G_{r\theta }d\theta dr+G_{rr}{\left(dr\right)}^{2}$$

in which $G_{\theta \theta }=(r+\zeta_{r}{)}^{2}{\left(1+\frac{\partial \zeta_{\theta }}{\partial \theta }\right)}^{2}+{\left(\frac{\partial \zeta_{r}}{\partial \theta }\right)}^{2}$, $G_{\tau \theta }=(r+\zeta_{r}{)}^{2}\left(1+\frac{\partial \zeta_{\theta }}{\partial \theta }\right)\frac{\partial \zeta_{\theta }}{\partial r}+\frac{\partial \zeta_{r}}{\partial \theta }\left(1+\frac{\partial \zeta_{r}}{\partial r}\right)$, $G_{rr}=(r+\zeta_{r}{)}^{2}{\left(\frac{\partial \zeta_{\theta }}{\partial r}\right)}^{2}+{\left(1+\frac{\partial \zeta_{r}}{\partial r}\right)}^{2}$.

Then, the line segment AB before and after deformation is analyzed, as shown in Figure 2b. According to the definition of strain, the tangential and normal strains can be obtained as follows:

(7a) $$\epsilon_{\theta }=\frac{ds_{\theta \theta }^{\prime }-rd\theta }{rd\theta }$$

(7b) $$\epsilon_{r}=\frac{ds_{rr}^{\prime }-dr}{dr}$$

And the shear strain is defined as the angle change in an infinitesimal element:

(7c) $$\epsilon_{r\theta }=\frac{\pi }{2}-\chi$$

Equation (9) can be obtained from the cosine theorem:

(8) $${\left(ds^{\prime }\right)}^{2}={\left(ds_{\theta \theta }^{\prime }\right)}^{2}+2cos\;\chi ds_{\theta \theta }^{\prime }ds_{rr}^{\prime }+{\left(ds_{rr}^{\prime }\right)}^{2}$$

By comparing Equations (6) and (9), substituting Equation (8) into them, and omitting the high-order infinitesimal, the strain expression can be obtained:

(9) $$\left\{\begin{array}{c} \epsilon_{\theta }\approx \frac{1}{r}\left(W+\frac{\partial V}{\partial \theta }\right)+\frac{1}{2r^{2}}{\left(V-\frac{\partial W}{\partial \theta }\right)}^{2}\\ \epsilon_{r}\approx \frac{\partial W}{\partial r}\\ \epsilon_{r\theta }\approx \frac{\partial V}{\partial r}+\frac{1}{r}\left(\frac{\partial W}{\partial \theta }-V\right) \end{array}\right.$$

According to the assumption of a thin ring, the Euler–Bernoulli beam theory can be used, that is, the cross-section plane perpendicular to the center line of the beam before deformation is still a plane after deformation (rigid cross-section assumption), and the plane of the cross-section after deformation is still perpendicular to the axis after deformation. Therefore, according to this theory, the only effective strain is the tangential strain $\epsilon_{\theta }$. The radial displacement W can be approximated as the displacement on the neutral layer of the ring, and the tangential displacement V varies linearly with the thickness of the ring:

(10) $$\begin{array}{c} W(\theta ,t)\approx w(\theta ,t)\\ V(\theta ,y,t)=v(\theta ,t)+y\cdot \beta (\theta ,t) \end{array}$$

where W,V are the radial and tangential displacements on the neutral layer, y is the distance from the point to the neutral layer, and β is the angle of rotation of the ring cross-section. Substituting Equation (11) into Equation (10) yields the following:

(11) $$\begin{array}{c} \epsilon_{\theta }=\frac{1}{R+y}\left(w+\frac{\partial v}{\partial \theta }+y\frac{\partial \beta }{\partial \theta }\right)+\frac{1}{2(R+y{)}^{2}}\left(\frac{\partial w}{\partial \theta }-v-y\beta \right)\\ \epsilon_{r\theta }=\frac{1}{R+y}\left(\frac{\partial w}{\partial \theta }-v+R\beta \right) \end{array}$$

According to the assumption of a thin circular ring, the average radius of the whole model is much larger than the thickness of the ring (R≫h), so R+y in the strain–displacement relation can be replaced by R. The shear strain can be considered to be zero, and therefore the cross-section angle of rotation can be obtained:

(12) $$\beta =\frac{1}{R}\left(v-\frac{\partial w}{\partial \theta }\right)$$

Substituting this into Equation (12) and neglecting the second-order terms with β and y, the final displacement–strain relationship relation can be obtained as follows:

(13) $$\epsilon_{\theta }=\frac{1}{R}\left(w+\frac{\partial v}{\partial \theta }\right)+\frac{y}{R^{2}}\left(\frac{\partial v}{\partial \theta }-\frac{\partial^{2}w}{\partial \theta^{2}}\right)+\frac{1}{2R^{2}}{\left(\frac{\partial w}{\partial \theta }-v\right)}^{2}$$

The first and third terms in Equation (13) can be understood as caused by the ring tensile deformation, and the second term is the representation related to the beam bending. In the subsequent analysis process, to be able to obtain a more simplified equation of motion and to introduce the inextensibility assumption here in advance, that is, in the deformation process, the neutral layer of the ring model in the circumferential length of the ring is kept constant, the circumferential strain is zero, and, at this time, the circumferential strain on the neutral layer can be simplified as follows:

(14) $$\epsilon_{\theta }=\frac{1}{R}\left(w+\frac{\partial v}{\partial \theta }\right)$$

From this, the displacement relationship under the assumption can be obtained:

(15) $$w=-\frac{\partial v}{\partial \theta }$$

### 2.2. Model Improvement

The flexible ring model regards each layer of the tire crown as a uniform homogeneous material when it is established, but in practice, the layers of the tire are different materials and thicknesses. Especially for the tread, it is a rubber specially configured for balancing wear, traction, handling, and rolling resistance, with a large thickness and stiffness. Therefore, the author believes that it is more appropriate to consider the elasticity and stiffness of the tread itself separately, and the ring in the flexible ring model is considered as the tire crown layer and its lower layers. This kind of treatment was first proposed by T. Akasaka [38] and used by K. Yamagishi [39] to solve the tire–road static contact problem. The improvement is to add a layer of radial spring distributed around the outer edge of the ring.

#### 2.2.1. Introduction of Tread Wear

**Figure 3:**
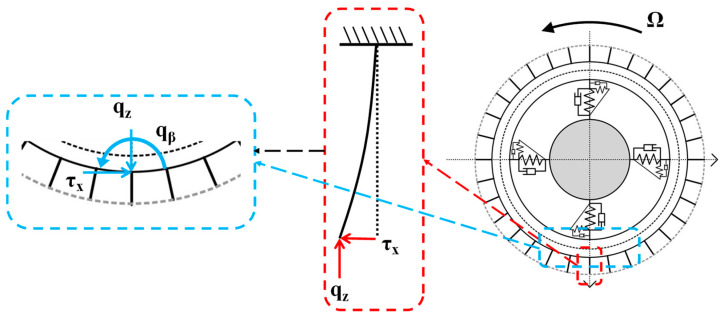
Schematic of the improved model: $q_{z}$ is the vertical force, $\tau_{x}$ is the longitudinal force, and $q_{\beta }$ is the longitudinal moment.

In this paper, for the tire tread part, the author improves it to a radial brush distributed along the circumference of the ring, and it has bending stiffness and length, similar to the brush model. The reason for this measure is that the brush model can make better use of the distributed load in the grounding area, and it can take tire wear into account in the model. As shown in Figure 3, it can be assumed that each brush bristle is subjected to tangential and vertical forces $\tau_{x}$ and $q_{z}$. At this point, assuming that the brush bristles are modeled as cantilever beams fixed to a ring at one end and analyzing the forces on them, we can obtain the forces on the fixed end of the brush bristles, i.e., the points corresponding to the flexible ring are subjected to reaction forces of equal magnitude, $\tau_{x}$ and $q_{z}$, as well as moments generated by the tangential forces, $q_{\beta }=l\cdot \tau_{x}$, where l is the length of the bristles, which is a variable that can be used to characterize the degree of wear of the tire when it is subjected to wear. This simplification allows tire wear to be taken into account in the subsequent analytical solution of the model.

#### 2.2.2. Introduction of the Pressure Distribution Function

##### Vertical Force Distribution

For the pressure distribution $q_{z}$ of the tire, this paper analyzes it using an arbitrary pressure distribution function in the Unitire model [40], which can obtain different shapes of the grounding pressure distribution by changing different parameters, which facilitates the analysis of the grounding state under different working conditions. In earlier studies [41,42,43], the pressure distribution was expressed as a symmetric parabolic distribution:

(16) $$q_{z}\left(x\right)=\frac{3F_{z}}{4a}\cdot \left[1-{\left(\frac{x}{a}\right)}^{2}\right]$$

**Figure 4:**
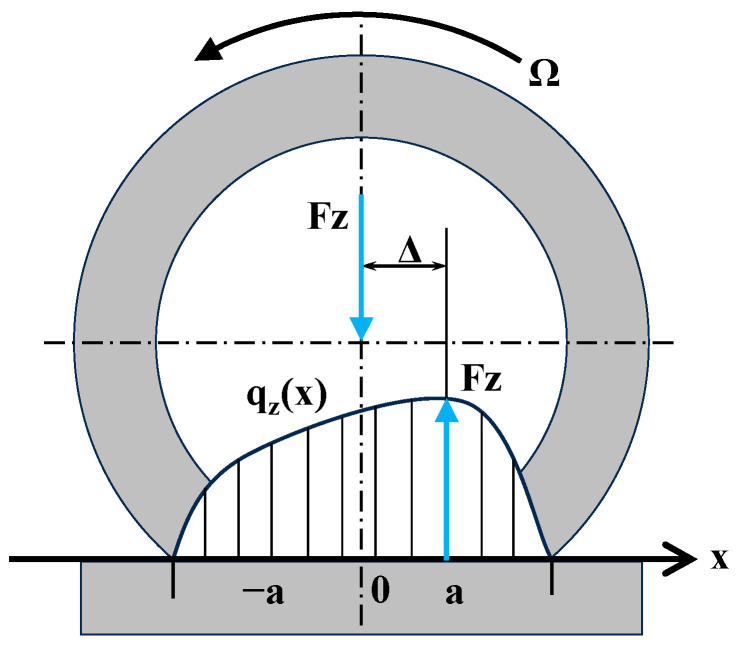
Schematic of Unitire’s uniform pressure distribution.

However, this pressure distribution function, with its unitary nature, cannot characterize the pressure distribution of some different working conditions. Instead, in the Unitire model, it is expressed by the following equation:

(17) $$q_{z}\left(x\right)=\frac{F_{z}}{2a}\cdot \eta \left(\frac{x}{a}\right)=\frac{F_{z}}{2a}\cdot \eta \left(u\right)$$

where a is the imprint half-length, u is the imprint relative coordinate, u=x/a, and η(u) is the dimensionless imprint pressure distribution function. η(u) should satisfy the following boundary conditions:

(18) $$\left\{\begin{array}{l} \eta \left(1\right)=\eta \left(-1\right)=0\\ \eta \left(u\right)\geq 0u\in \left[-1,1\right]\\ \eta \left(u\right)=0u\notin \left[-1,1\right]\\ \int_{-1}^{1}\eta \left(u\right)du=2\\ \int_{-1}^{1}\eta \left(u\right)udu=2\frac{\Delta }{a} \end{array}\right.$$

where Δ is the offset of the vertical load, as shown in Figure 4.

For the dimensionless imprinted pressure distribution function η(u), Guo [40,44] gives the following unified expression:

(19) $$\eta \left(u\right)=A\left(1-u^{2n}\right)\left(1+\lambda \cdot u^{2n}\right)\left(1-B\cdot u\right)$$

Based on the boundary conditions, the expressions for A and B can be obtained:

(20a) $$A=\frac{\left(2n+1\right)\left(4n+1\right)}{2n\left(4n+1+\lambda \right)}$$

(20b) $$B=-\frac{3\left(2n+3\right)\left(4n+3\right)\left(4n+1+\lambda \right)}{\left(2n+1\right)\left(4n+1\right)\left(4n+3+3\lambda \right)}\frac{\Delta }{a}$$

The parameter n is the uniformity factor, which affects the uniformity of the pressure distribution. λ is the concave–convex factor, which affects the concave–convex shape of the curve. Δ/a is a skew factor, which affects the position distribution of the distributed pressure. These three factors are related to variables such as vertical load, tire pressure, and rolling speed. Through the expression, any form of pressure distribution in the tire contact patch can be described.

##### Longitudinal Distribution of Forces

The longitudinal forces are formulated using the brush model, where the tangential and vertical problems are assumed to be decoupled in the brush theory, which suggests that the vertical pressure distribution is not affected by the shear stresses inside the contact patch [45]. According to Pacejka [46], the basic equations controlling the contact mechanics of tire pavement can be formulated using a simple Coulomb friction model as follows:

(21) $$\tau_{x}\left(\varphi \right)=\left\{\begin{array}{l} \left.\pm (a-x(\varphi ))k_{x}\varepsilon_{x}\right.,\left|(a-x(\varphi ))k_{x}\varepsilon_{x}\right|\leq \mu_{s}q_{z}(\varphi )\\ \pm \mu_{d}q_{z}(\varphi )sgn(\varepsilon_{x}),\;\;elsewhere \end{array}\right.$$

in which

$$\varepsilon_{x}=\frac{V_{x}-\Omega R}{V_{x}}\;a=\left|R*\sin\left(\varphi_{f}\right)\right|\;x\left(\varphi \right)=R*\sin\;(\varphi )$$

Since there are adhesion and slip areas in the grounding region when the actual tire is rolling, the distribution of longitudinal force needs to consider the tire slip condition. The symbols + and − denote the braking and acceleration conditions, respectively. $\varepsilon_{x}$ is the slip rate of the tire, whose expression $V_{x}$ is the forward speed of the tire, and ΩR denotes the linear speed of the wheel rotation. In the adhesion area, the longitudinal force at different positions φ is expressed as $(a-x(\varphi ))k_{x}\varepsilon_{x}$, where $k_{x}$ is the longitudinal stiffness of the tread. In the slip area, the longitudinal force is expressed as kinetic friction.

According to Section 2.2.1, the distributed moment $q_{\beta }=l\cdot \tau_{x}$, which is an approximate distributed moment expression, can be obtained by substituting the above longitudinal force expression, at which point, if the tire has a wear condition, it can be characterized by changing the length of the brush l for subsequent analysis.

## 3. Model Analysis and Solution

If this model is to be applied in a strain-based intelligent tire system, further exploitation of the previous strain–displacement relationship is required. In the following section, the model’s equations of motion are obtained by performing an energy method analysis of the tire system.

**Figure 5:**
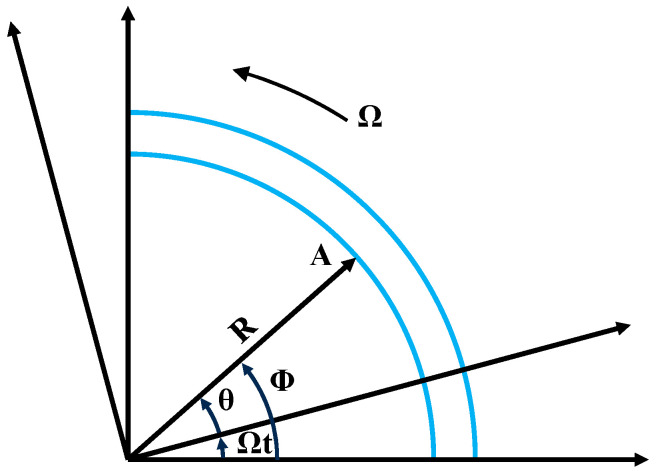
The coordinate systems (Ωt: Wheel Rotation Angle; θ: angle of point A in the rotated coordinate system; ϕ: angle of point A in the fixed coordinate system.).

First of all, a suitable coordinate system is established for the tire system, assuming that the wheel rolls forward freely with an average angular velocity Ω. With the center of the wheel as the origin, the forward direction of the wheel is the *x*-axis, and the upward direction perpendicular to the road surface is the *z*-axis, so the wheel has only three degrees of freedom. In addition, to be able to better study the motion of the wheel in the rolling process, a rotating coordinate system is also established, as shown in Figure 5. Its relationship with the fixed coordinate system is as follows:

(22) $$\left\{\begin{array}{l} x=x^{*}cos\;(\Omega t)-z^{*}sin\;(\Omega t)\\ z=x^{*}sin\;(\Omega t)+z^{*}cos\;(\Omega t)\\ \phi =\theta +\Omega t \end{array}\right.$$

Because the motion of the tire system is a complex nonlinear process but its motion process is a process of time history, the Hamilton principle is used to analyze it. The Hamilton principle can be expressed as a system with conservative force at the same time and the same starting and ending positions. In all possible motions, the real motion is to make the Hamiltonian functional have a stable value, which is expressed as follows:

(23) $$\delta H=\delta \int_{t_{0}}^{t_{1}}L\left(q_{s},{\dot{q}}_{s},t\right)dt=0$$

where L is the Lagrangian function L=T−V.

For the tire system, in addition to the kinetic energy and potential energy in the Lagrange function, it also has the work carried out by the external force. Based on the D’Alembert principle and the principle of virtual work in the generalized coordinate system, it is only necessary to add the work carried out by the external force to the Lagrange equation of the system when calculating the Hamiltonian functional. The Hamilton principle is still applicable, and the motion law of the system can be obtained. At the same time, to keep the motion equation of the model linear, it is assumed that the wheel angular velocity Ω has a slight change ${\dot{\theta }}_{r}$.

### 3.1. Energy Equation

#### 3.1.1. Kinetic Energy

The volume infinitesimal of the ring is taken to integrate its kinetic energy:

(24) $$T_{1}=b\int_{0}^{2\pi }\int_{-\frac{h}{2}}^{\frac{h}{2}}\frac{1}{2}\rho R{|\dot{\vec{\gamma }}|}^{2}dyd\theta$$

in which

(25) $$\vec{\gamma }=\left(R+w\right){\vec{n}}_{r}+v{\vec{n}}_{\theta }$$

$\vec{\gamma }$ is the coordinate of the point on the ring after deformation. ${\vec{n}}_{r}$ and ${\vec{n}}_{\theta }$ represent the radial and tangential unit components in the undeformed state, respectively. The time variation is related to the angle. Therefore, when calculating the velocity vector of this point, the derivative formula of multiplication should be used. Thus, it is obtained:

(26) $$\dot{\vec{\gamma }}=\left(\dot{w}-v\Omega \right){\vec{n}}_{r}+\left[\dot{v}+\left(R+w\right)\Omega \right]{\vec{n}}_{\theta }$$

In addition to the point on the ring having kinetic energy, the wheel itself also has kinetic energy:

(27) $$T_{2}=\frac{1}{2}\left[m\left({\dot{x}}^{2}+{\dot{z}}^{2}\right)+I_{r}{\left(\Omega +{\dot{\theta }}_{r}\right)}^{2}\right]$$

where m is the mass of the wheel, x and z are the displacements at the center of the wheel, $I_{r}$ is the moment of inertia of the wheel, and ${\dot{\theta }}_{r}$ is the change value of the average angular velocity Ω at a certain moment. Therefore, according to Equations (24) and (28), the kinetic energy of the whole system in the rotating coordinate system is

(28) $$T=\int_{0}^{2\pi }\frac{1}{2}\rho AR\left[{\left(\dot{w}-v\Omega \right)}^{2}+{\left(\dot{v}+\left(R+w\right)\Omega \right)}^{2}\right]d\theta +\frac{1}{2}\left[m({\left({\dot{x}}^{*}-\Omega z^{*}\right)}^{2}+{\left({\dot{z}}^{*}+\Omega x^{*}\right)}^{2})+I_{r}{\left(\Omega +{\dot{\theta }}_{r}\right)}^{2}\right]$$

#### 3.1.2. Potential Energy

During the deformation of the tire, a part of the potential energy is stored in the system in the form of stress and strain. Therefore, according to the strain value of a certain point of the tire obtained in the second chapter, the double integral can be used to characterize the strain energy of the ring. However, because the system is also affected by the air pressure in the tire and the centrifugal force when the system is moving, a pre-stress is defined to represent the stress generated by the two parts:

(29) $$\sigma_{\theta }^{0}=\frac{\left(p_{0}bR+\rho AR^{2}\Omega^{2}\right)}{A}$$

where $p_{0}$ is the tire pressure, b is the width of the ring, R is the radius of the neutral layer position, ρ is the ring density, A=bh is the cross-sectional area of the ring, and Ω is the angular velocity of the ring rotation. This part is related to the tire geometric parameters and tire pressure, so it can be regarded as a constant.

The strain energy of the ring model is as follows:

(30) $$V_{1}=b\int_{0}^{2\pi }\int_{-\frac{h}{2}}^{\frac{h}{2}}\left[\frac{1}{2}\sigma_{\theta }\epsilon_{\theta }+\sigma_{\theta }^{0}\epsilon_{\theta }\right]Rdyd\theta$$

Assuming that Hooke’s theorem applies to the stress growth process of the ring, we can substitute Equation (14) into the above equation.

(31) $$\begin{array}{c} V_{1}=\int_{0}^{2\pi }\frac{1}{2}\left\{2\sigma_{\theta }^{0}A\left(w+\frac{\partial v}{\partial \theta }\right)+\frac{\sigma_{\theta }^{0}A}{R}{\left(v-\frac{\partial w}{\partial \theta }\right)}^{2}\right.\left.+\frac{E}{R}\left[A{\left(w+\frac{\partial v}{\partial \theta }\right)}^{2}+\frac{I}{R^{2}}{\left(\frac{\partial v}{\partial \theta }-\frac{\partial^{2}w}{\partial \theta^{2}}\right)}^{2}\right]\right\}\;d\theta \end{array}$$

where E is the Young’s modulus of the ring, and $I=\frac{bh^{3}}{12}$ is the moment of inertia of the ring section. There is also a portion of the potential energy in the system that is stored in the spring base of the model:

(32) $$V_{2}=\int_{0}^{2\pi }\frac{1}{2}\left.\begin{array}{c} \left[k_{v}{\left(v+x^{*}\sin\;\theta -z^{*}\cos\;\theta -R\theta_{r}\right)}^{2}\right. \end{array}\right.+\left.k_{w}{\left(w-x^{*}\cos\;\theta -z^{*}\sin\;\theta \right)}^{2}\right]Rd\theta$$

where $\theta_{r}$ is the angular displacement caused by the change in the angular velocity; ${\dot{\theta }}_{r}$, $x^{*}$, and $z^{*}$ are the displacement at the center of the wheel after the deformation of the tire during the rotation of the wheel. The potential energy of the whole system is as follows:

(33) $$\begin{array}{ll} V= & \int_{0}^{2\pi }\frac{1}{2}\left.\begin{array}{c} \left\{2\sigma_{\theta }^{0}A\left(w+\frac{\partial v}{\partial \theta }\right)+\frac{\sigma_{\theta }^{0}A}{R}{\left(v-\frac{\partial w}{\partial \theta }\right)}^{2}\left.+\frac{E}{R}\left[A{\left(w+\frac{\partial v}{\partial \theta }\right)}^{2}+\frac{I}{R^{2}}{\left(\frac{\partial v}{\partial \theta }-\frac{\partial^{2}w}{\partial \theta^{2}}\right)}^{2}\right]\right\}d\theta \right. \end{array}\right.\\ & +\int_{0}^{2\pi }\frac{1}{2}[k_{w}(v+x^{*}\sin\;\theta -z^{*}cos\;\theta -R\theta_{r}{)}^{2}+k_{w}(w-x^{*}cos\;\theta -z^{*}sin\;\theta {)}^{2}]Rd\theta \end{array}$$

#### 3.1.3. Virtual Work of External Forces

During the movement of the wheel, it is subjected to the vertical force, $f_{z}$ longitudinal force $f_{x}$, and torque T from the axle, as well as the distributed force $q_{w}$, $q_{v}$, and distributed torque $q_{\beta }$ from the ground. These external forces cause the deformation of the tire. The virtual work carried out by these external forces over the virtual displacements is as follows:

(34) $$\delta E_{1}=\int_{0}^{2\pi }\left[q_{w}\delta w+q_{v}\delta v+q_{\beta }\delta \beta \right]Rd\theta +f_{x*}\delta z^{*}+f_{z*}\delta z^{*}+T\delta \theta_{r}$$

Based on the preceding, $\beta =\frac{1}{R}\left(v-\frac{\partial w}{\partial \theta }\right)$.

According to the properties of variation,

(35) $$\delta \beta =\frac{1}{R}\left(\delta v-\frac{\partial }{\partial \theta }\delta w\right)$$

Substituting (35) into Equation (34) yields the following:

(36) $$E_{1}=\int_{0}^{2\pi }\left[\left(q_{w}+\frac{1}{R}\cdot \frac{\partial q_{\beta }}{\partial \theta }\right)\delta w+\left(q_{v}+\frac{1}{R}\cdot q_{\beta }\right)\delta v\right]Rd\theta +f_{x*}\delta z^{*}+f_{z*}\delta z^{*}+T\delta \theta_{r}$$

For the imaginary displacements δw and δv, which are subject to the action of the air pressure inside the tire since the tire pressure always acts in the direction perpendicular to the inner liner of the tire, the unit normal vector after the ring deformation is taken to be the direction of the action of the tire pressure:

(37) $$\vec{n}\approx \left[1+\frac{1}{R}\left(\frac{\partial v}{\partial \theta }+w\right)\right]{\vec{n}}_{r}-\frac{1}{R}\left(\frac{\partial w}{\partial \theta }-v\right){\vec{n}}_{\theta }$$

Therefore, the work carried out by the tire pressure is as follows:

(38) $$\delta E_{2}=\int_{0}^{2\pi }p_{0}b\left[\left(1+\frac{1}{R}\left(\frac{\partial v}{\partial \theta }+w\right)\right)\delta w\right.\left.-\frac{1}{R}\left(\frac{\partial w}{\partial \theta }-v\right)\delta v\right]Rd\theta$$

Then, the total external virtual work can be obtained: $\delta E=\delta E_{1}+\delta E_{2}$. The Lagrangian function can be expressed as follows:

(39) L=T−V+E

#### 3.1.4. Solution of Motion Equation

To sum up the analysis of virtual work and energy, the Hamiltonian principle can be expressed as follows:

(40) $$\delta H=\delta \int_{t_{0}}^{t_{1}}\left(T-V+E\right)dt=0$$

The Euler–Lagrange equation corresponding to the generalized coordinates of the ring model can be obtained by solving the variation according to the above formula.

(41) $$\left\{\begin{array}{c} \begin{array}{l} \frac{\partial }{\partial t}\frac{\partial L}{\partial \dot{w}}+\frac{\partial }{\partial \theta }\frac{\partial L}{\partial w^{\prime }}-\frac{\partial^{2}}{\partial \theta^{2}}\frac{\partial L}{\partial w^{\prime \prime }}-\frac{\partial L}{\partial w}=\int_{0}^{2\pi }\left[\left(q_{w}+\frac{q_{\beta }\prime }{R}\right)+p_{0}b\left(1+\frac{1}{R}\left(v^{\prime }+w\right)\right)\right]Rd\theta \\ \frac{\partial }{\partial t}\frac{\partial L}{\partial \dot{v}}+\frac{\partial }{\partial \theta }\frac{\partial L}{\partial v^{\prime }}-\frac{\partial L}{\partial v}=\int_{0}^{2\pi }\left[\left(q_{v}+\frac{q_{\beta }}{R}\right)-\frac{1}{R}\left(w^{\prime }-v\right)p_{0}b\right]Rd\theta \\ \frac{\partial }{\partial t}\frac{\partial L}{\partial {\dot{\theta }}_{r}}-\frac{\partial L}{\partial \theta_{r}}=T\\ \frac{\partial }{\partial t}\frac{\partial L}{\partial {\dot{x}}^{*}}-\frac{\partial L}{\partial x^{*}}=f_{x*}\\ \frac{\partial }{\partial t}\frac{\partial L}{\partial {\dot{z}}^{*}}-\frac{\partial L}{\partial z^{*}}=f_{z*} \end{array} \end{array}\right.$$

The dot (˙) on the symbol represents the differential of the term to time, and (‘) represents the differential of the term to θ.

Substituting Equation (39) into Equation (41), and separating the expression, we can obtain the following:

(42a) $$q_{w}+\frac{q_{\beta }\prime }{R}=\frac{EI}{R^{4}}\left(\frac{\partial^{4}w}{\partial \theta^{4}}-\frac{\partial^{3}v}{\partial \theta^{3}}\right)+\frac{EA}{R^{2}}\left(w+\frac{\partial v}{\partial \theta }\right)+\frac{\sigma_{\theta }^{0}A}{R^{2}}\left(\frac{\partial v}{\partial \theta }-\frac{\partial^{2}w}{\partial \theta^{2}}\right)-\frac{p_{0}b}{R}\left(\frac{\partial v}{\partial \theta }+w\right)+\frac{\sigma_{\theta }^{0}A}{R}\;+k_{w}\left(w-x^{*}cos\theta -z^{*}sin\theta \right)+\rho A\left(\ddot{w}-2\Omega \dot{v}-\Omega^{2}w-\Omega^{2}R\right)$$

(42b) $$\begin{array}{c} q_{v}+\frac{q_{\beta }}{R}=\frac{EI}{R^{4}}\left(\frac{\partial^{3}w}{\partial \theta^{3}}-\frac{\partial^{2}v}{\partial \theta^{2}}\right)-\frac{EA}{R^{2}}\left(\frac{\partial w}{\partial \theta }+\frac{\partial^{2}v}{\partial \theta^{2}}\right)+\frac{\sigma_{\theta }^{0}A}{R^{2}}\left(v-\frac{\partial w}{\partial \theta }\right)+\frac{p_{0}b}{R}\left(\frac{\partial w}{\partial \theta }-v\right)\\ +k_{v}\left(v+x^{*}\sin\theta -z^{*}\cos\theta -R\theta_{r}\right)+\rho A\left(\ddot{v}+2\Omega \dot{w}-\Omega^{2}v\right) \end{array}$$

(42c) $$T=I_{r}{\ddot{\theta }}_{r}+2\pi k_{v}R^{3}\theta_{r}-R^{2}\left.\int_{0}^{2\pi }k_{v}v\right.d\theta$$

(42d) $$f_{x^{*}}=m\left({\ddot{x}}^{*}-2\Omega {\dot{z}}^{*}-\Omega^{2}x^{*}\right)+\pi R\left(k_{w}+k_{v}\right)x^{*}-R\int_{0}^{2\pi }\left(k_{w}\mathrm{wcos}\theta -k_{v}\mathrm{vsin}\theta \right)d\theta$$

(42e) $$f_{z^{*}}=m\left({\ddot{z}}^{*}+2\Omega {\dot{x}}^{*}-\Omega^{2}z^{*}\right)+\pi R\left(k_{w}+k_{v}\right)z^{*}-R\int_{0}^{2\pi }\left(k_{w}\mathrm{wsin}\theta +k_{v}\mathrm{vcos}\theta \right)d\theta$$

According to the inextensibility assumption in the second chapter, the motion equation can be further simplified. At the same time, to keep the variable order consistent, the derivative of θ is first obtained, and then the sum is obtained:

(43a) $$\begin{array}{ll} q_{v}+\frac{\partial q_{w}}{\partial \theta }+\frac{1}{R}\left(q_{\beta }+\frac{\partial^{2}q_{\beta }}{\partial \theta^{2}}\right)= & -\frac{EI}{R^{4}}\left(\frac{\partial^{2}v}{\partial \theta^{2}}+2\frac{\partial^{4}v}{\partial \theta^{4}}+\frac{\partial^{6}v}{\partial \theta^{6}}\right)+\frac{\sigma_{\theta }^{0}A}{R^{2}}\left(v+2\frac{\partial^{2}v}{\partial \theta^{2}}+\frac{\partial^{4}v}{\partial \theta^{4}}\right)-\frac{p_{0}b}{R}\left(v+\frac{\partial^{2}v}{\partial \theta^{2}}\right)-k_{w}\frac{\partial^{2}v}{\partial \theta^{2}}\\ & +k_{v}\left(v-R\theta_{r}\right)+\left(k_{w}+k_{v}\right)\left(x^{*}sin\;\theta -z^{*}cos\;\theta \right)+\rho A\left[\ddot{v}-\frac{\partial^{2}\ddot{v}}{\partial \theta^{2}}-4\Omega \frac{\partial \dot{v}}{\partial \theta }+\Omega^{2}\left(\frac{\partial^{2}v}{\partial \theta^{2}}-v\right)\right] \end{array}$$

(43b) $$T=I_{r}{\ddot{\theta }}_{r}+2\pi k_{v}R^{3}\theta_{r}-R^{2}\left.\int_{0}^{2\pi }k_{v}v\right.d\theta$$

(43c) $$f_{x^{*}}=m\left({\ddot{x}}^{*}-2\Omega {\dot{z}}^{*}-\Omega^{2}x^{*}\right)+\pi R\left(k_{w}+k_{v}\right)x^{*}-R\int_{0}^{2\pi }\left(-k_{w}\frac{\partial v}{\partial \theta }\cos\theta -k_{v}\mathrm{vsin}\theta \right)d\theta$$

(43d) $$f_{z^{*}}=m\left({\ddot{z}}^{*}+2\Omega {\dot{x}}^{*}-\Omega^{2}z^{*}\right)+\pi R\left(k_{w}+k_{v}\right)z^{*}-R\int_{0}^{2\pi }\left(-k_{w}\frac{\partial v}{\partial \theta }\sin\theta +k_{v}\mathrm{vcos}\theta \right)d\theta$$

At this point, the relationship between the generalized force and the displacement in the model is obtained, which can be used for the subsequent solution of the strain.

### 3.2. Displacement Expression Solution

#### 3.2.1. Tire Vibration Equation

In the previous section, it is known that the tire itself can be regarded as a vibration system, so it can be assumed that the vibration equation of the system is as follows:

(44) $$M\ddot{x}+C\dot{x}+Kx=F$$

where M is the mass matrix, C is the damping matrix, K is the stiffness matrix, and F is the external force.

Because the vibration mode of the tire is more complex, we can use the modal expansion method to decompose and simplify the dynamic problem by using the inherent mode of the system. It is described that the response of the system to any external excitation force can be expressed as a weighted sum of the inherent modal shapes of the system. Therefore, the tangential displacement of the ring can be expressed by Fourier expansion as follows:

(45) $$v\left(\theta ,t\right)=\sum_{n=0}^{+\infty }\left[a_{n}\left(t\right)cos\left(n\theta \right)+b_{n}\left(t\right)sin\left(n\theta \right)\right]$$

where $a_{n}\;and\;b_{n}$ are the quantities related to the natural frequency and time of the n-order mode, which can be used to represent the modal participation factor.

This section mainly studies tire displacement under external excitation in the contact area between the tire and the road surface, so the degree of freedom of the tire translation is constrained, that is, ${x=z=\theta }_{r}=0$. Substituting the displacement expression into Equation (45), we can obtain the following:

(46) $$\left.\left[\begin{array}{cc} m_{n}\cos\left(n\theta \right) & 0\\ 0 & m_{n}\sin\left(n\theta \right) \end{array}\right.\right]\left\{\begin{array}{c} {\ddot{a}}_{n}\\ {\ddot{b}}_{n} \end{array}\right\}+\left[\begin{array}{cc} 0 & g_{n}\sin\left(n\theta \right)\\ -g_{n}\cos\left(n\theta \right) & 0 \end{array}\right]\left\{\begin{array}{c} {\dot{a}}_{n}\\ {\dot{b}}_{n} \end{array}\right\}+\left[\begin{array}{cc} k_{n}\cos\left(n\theta \right) & 0\\ 0 & k_{n}\sin\left(n\theta \right) \end{array}\right]\left\{\begin{array}{c} a_{n}\\ b_{n} \end{array}\right\}=f_{n}$$

where $m_{n}=\rho A(1+n^{2})$, $g_{n}=-4\rho An\Omega$, $k_{n}=\left(\frac{EI}{R^{4}}n^{2}+\frac{\sigma_{\theta }^{0}A}{R^{2}}\right)(1-n^{2}{)}^{2}-\frac{p_{0}b}{R(1-n^{2})}+k_{v}+k_{w}n^{2}-\rho A\Omega^{2}(1+n^{2})-\rho A\Omega^{2}(1+n^{2})$.

The expression is defined in the rotating coordinate system. From the previous text, it can be transformed into the fixed coordinate system by ϕ=θ+Ωt. In the fixed coordinate system, the corresponding coefficient value becomes the following: $m_{n}=\rho A(1+n^{2})$, $g_{n}=2n\rho A\Omega (n^{2}-1)$, $k_{n}=\left(\frac{EI}{R^{4}}n^{2}+\frac{\sigma_{\theta }^{0}A}{R^{2}}\right)(1-n^{2}{)}^{2}-\frac{p_{0}b}{R(1-n^{2})}+k_{v}+k_{w}n^{2}-\rho A\Omega^{2}(1-n^{2}{)}^{2}$.

To decompose the vibration matrix equation into independent equations and eliminate the trigonometric function, we perform Fourier series expansion on the external excitation $f_{n}$ and obtain the following:

(47) $$f_{n}=\sum_{n=1}^{\infty }\left(\xi_{n}\cos\left(n\theta \right)+\eta_{n}\sin\left(n\theta \right)\right)$$

Its Fourier coefficient is as follows:

(48) $$\left[\begin{array}{c} \xi_{n}\\ \eta_{n} \end{array}\right]=\left[\begin{array}{c} \frac{1}{\pi }\int_{0}^{2\pi }(q_{v}+\frac{\partial q_{w}}{\partial \theta }+\frac{1}{R}(q_{\beta }+\frac{\partial^{2}q_{\beta }}{\partial \theta^{2}}))cos\;n\theta d\theta \\ \frac{1}{\pi }\int_{0}^{2\pi }\left(q_{v}+\frac{\partial q_{w}}{\partial \theta }+\frac{1}{R}\left(q_{\beta }+\frac{\partial^{2}q_{\beta }}{\partial \theta^{2}}\right)\right)\sin n\theta d\theta \end{array}\right]$$

Subsequently, an easily solvable vibration equation is obtained:

(49) $$\left.\left[\begin{array}{cc} m_{n} & 0\\ 0 & m_{n} \end{array}\right.\right]\left\{\begin{array}{c} {\ddot{a}}_{n}\\ {\ddot{b}}_{n} \end{array}\right\}+\left[\begin{array}{cc} 0 & g_{n}\\ -g_{n} & 0 \end{array}\right]\left\{\begin{array}{c} {\dot{a}}_{n}\\ {\dot{b}}_{n} \end{array}\right\}+\left[\begin{array}{cc} k_{n} & 0\\ 0 & k_{n} \end{array}\right]\left\{\begin{array}{c} a_{n}\\ b_{n} \end{array}\right\}=\left\{\begin{array}{c} \xi_{n}\\ \eta_{n} \end{array}\right\}$$

Since the tire has a damping effect in practice and has a great influence on the vibration analysis of the tire, the damping factor can be added to the damping matrix to characterize it. The vibration equation with damping can be written as follows:

(50) $$\left.\left[\begin{array}{cc} m_{n} & 0\\ 0 & m_{n} \end{array}\right.\right]\left\{\begin{array}{c} {\ddot{a}}_{n}\\ {\ddot{b}}_{n} \end{array}\right\}+\left[\begin{array}{cc} c_{n} & g_{n}\\ -g_{n} & c_{n} \end{array}\right]\left\{\begin{array}{c} {\dot{a}}_{n}\\ {\dot{b}}_{n} \end{array}\right\}+\left[\begin{array}{cc} k_{n} & 0\\ 0 & k_{n} \end{array}\right]\left\{\begin{array}{c} a_{n}\\ b_{n} \end{array}\right\}=\left\{\begin{array}{c} \xi_{n}\\ \eta_{n} \end{array}\right\}$$

It is described by gong [37] that $c_{n}=2\lambda \sqrt{m_{n}k_{n}}$, where λ is the dimensionless damping coefficient, which generally has different values for different modals and is assumed to be the same for all the modals in this study.

#### 3.2.2. Tire Response to Concentrated Load

After the above analysis, the only unknown variables in the equation are $q_{\nu }$, $q_{w}$, and $q_{\beta }$, so this section analyzes the stress state of the tire in detail.

First, consider the case where the force and torque are concentrated on the tire, that is, all the external forces are applied to a point on the tread, which is represented by the Dirac δ function:

(51) $$\begin{array}{l} q_{w}\left(\theta ,t\right)=-Q_{w}\delta \left(\phi -\phi_{0}\right)=-Q_{w}\delta \left[\theta -\left(\phi_{0}-\Omega t\right)\right]\\ q_{\nu }\left(\theta ,t\right)=Q_{\nu }\delta \left(\phi -\phi_{0}\right)=Q_{\nu }\delta \left[\theta -\left(\phi_{0}-\Omega t\right)\right]\\ q_{\beta }\left(\theta ,t\right)=Q_{\beta }\delta \left(\phi -\phi_{0}\right)=Q_{\beta }\delta \left[\theta -\left(\phi_{0}-\Omega t\right)\right] \end{array}$$

$Q_{w},{\;Q}_{v},and\;Q_{\beta }$ are the radial external force, tangential external force, and external torque at a point φ_0, which can be expressed as $\phi_{0}-\Omega t$ in the rotating coordinate system. Substituting the formula into it, we can obtain the following:

(52a) $$\xi_{n}=\frac{1}{\pi }\left[\begin{array}{c} \left(Q_{\nu }+\left(1-n^{2}\right)Q_{\beta }\right)\cos\left(n\left(\phi_{0}-\Omega t\right)\right) \end{array}\right.\left.+nQ_{w}\sin\left(n\left(\phi_{0}-\Omega t\right)\right)\right]$$

(52b) $$\zeta_{n}=\frac{1}{\pi }\left[\begin{array}{c} \left(Q_{\nu }+\left(1-n^{2}\right)Q_{\beta }\right)sin\left(n\left(\phi_{0}-\Omega t\right)\right) \end{array}\right.\left.-nQ_{w}\cos\left(n\left(\phi_{0}-\Omega t\right)\right)\right]$$

The integration of the Dirac function makes use of its screening. Substitute the above equation into the vibration equation, and assume that $a_{n}$ and $b_{n}$ have the same form as $\xi_{n}$ and $\zeta_{n}$, which is as follows:

$$a_{n}=Acos\left(n\left(\phi_{0}-\Omega t\right)\right)+Bsin(n\left(\phi_{0}-\Omega t\right))\;b_{n}=Ccos\left(n\left(\phi_{0}-\Omega t\right)\right)+Dsin(n\left(\phi_{0}-\Omega t\right))$$

By solving the variables A, B, C, and D, the specific expression of the tangential displacement corresponding to a certain point can be solved.

(53) $$v\left(\phi \right)=\sum_{n=0}^{\infty }\left[\begin{array}{c} A_{n1}\left(Q_{\nu }+\left(1-n^{2}\right)Q_{\beta }\right)\cos\left(n\left(\phi_{0}-\phi +\gamma_{n}\right)\right)\left.+A_{n2}Q_{w}\sin\left(n\left(\phi_{0}-\phi +\gamma_{n}\right)\right)\right] \end{array}\right.$$

where $A_{n1}=\frac{1}{\pi \sqrt{{\left(M_{n}-G_{n}\right)}^{2}+{\left(C_{n}\right)}^{2}}}$, $A_{n2}=nA_{n1}$, $M_{n}=k_{n}-(n\Omega {)}^{2}m_{n}$, $G_{n}=(n\Omega )g_{n}$, and $C_{n}=(n\Omega )c_{n}$, and $\gamma_{n}$ is the correlation of the auxiliary angle formula in the solving process. $n\gamma_{n}={tan}^{-1}\;\left(\frac{C_{n}}{M_{n}-G_{n}}\right)$.

#### 3.2.3. Tire Response to Distributed Load

**Figure 6:**
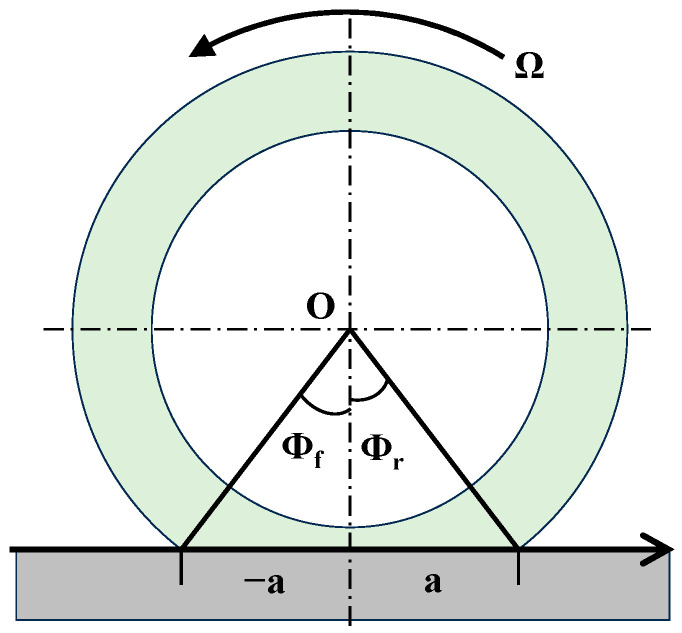
Grounding angle diagram.

Now consider the tire in the grounding area, that is, the area between the front grounding angle $\phi_{f}$ and the rear grounding angle $\phi_{r}$, as shown in Figure 6.

Assuming that the contact force acting on the tire is a distributed form, $q_{\nu }(\phi_{0})$, $q_{w}(\phi_{0})$, $q_{\beta }(\phi_{0})(\phi_{f}\leq \phi_{0}\leq \phi_{r})$; then, integrate the excitation of the concentrated force along the contact length to obtain the total displacement of the tire tread, which is as follows:

(54) $$\begin{array}{c} v\left(\phi \right)=\int_{\phi_{f}}^{\phi_{r}}\sum_{n=0}^{\infty }\left[A_{n1}\left(q_{\nu }\left(\phi_{0}\right)+\left(1-n^{2}\right)q_{\beta }\left(\phi_{0}\right)\right)\right.\cos\left.\left(n\left(\phi_{0}-\phi +\gamma_{n}\right)\right)+A_{n2}q_{w}\left(\phi_{0}\right)\sin\left(n\left(\phi_{0}-\phi +\gamma_{n}\right)\right)\right]d\phi_{0} \end{array}$$

Expand the integral formula to obtain

(55) $$v\left(\phi \right)=\sum_{n=0}^{\infty }\left.\left[\left(\alpha_{n1}A_{n1}+\alpha_{n3}A_{n2}\right)\sin\left(n\left(\phi -\gamma_{n}\right)\right)+\left(\alpha_{n2}A_{n1}-\alpha_{n4}A_{n2}\right)\cos\left(n\left(\phi -\gamma_{n}\right)\right)\right]\right.$$

At the same time, according to the inextensibility assumption, it can be seen that

(56) $$\begin{array}{ll} w\left(\phi \right) & =-\frac{\partial \nu }{\partial \theta }\\ & =-\sum_{n=0}^{\infty }n[\left(\alpha_{n1}A_{n1}+\alpha_{n3}A_{n2}\right)\cos\left(n\left(\phi -\gamma_{n}\right)\right)-(\alpha_{n2}A_{n1}-\alpha_{n4}A_{n2})sin(n(\phi )-\gamma_{n}))] \end{array}$$

where

$$\alpha_{n1}=\int_{\phi_{f}}^{\phi_{r}}(q_{v}(\phi )+(1-n^{2})\frac{q_{\beta }(\phi )}{R})sin\;(n\phi )d\phi \;\alpha_{n2}=\int_{\phi_{f}}^{\phi_{r}}(q_{v}(\phi )+(1-n^{2})\frac{q_{\beta }(\phi )}{R})cos\;(n\phi )d\phi \;\alpha_{n3}=\int_{\phi_{f}}^{\phi_{r}}q_{w}(\phi )cos\;(n\phi )d\phi \;\alpha_{n4}=\int_{\phi_{f}}^{\phi_{r}}q_{w}(\phi )sin\;(n\phi )d\phi$$

For the radial distribution force and tangential distribution force, the vertical pressure and the tire longitudinal distribution force in Section 2 can be used to decompose the force. Because the angle between the flexible ring and the ground is very small in the grounding area, the contribution of the vertical force in the tangential direction is very small, so it is neglected.

Thus, we can obtain the following:

(57a) $$q_{w}\left(\phi \right)=q_{z}\left(\phi \right)\cos\left(\phi \right)+\tau_{x}\left(\phi \right)\sin\left(\phi \right)$$

(57b) $$q_{v}\left(\phi \right)=\tau_{x}\left(\phi \right)\cos\left(\phi \right)$$

(57c) $$q_{\beta }\left(\phi \right)=l\cdot \tau_{x}\left(\phi \right)$$

By substituting the above formula into Equations (55) and (56), the corresponding specific radial and tangential displacements can be obtained.

#### 3.2.4. Circumferential Strain Equation

In the actual intelligent tire, the installation position of the strain gauge is not in the tread. With the current level of tire manufacturing, it is also more difficult to install the sensor between the belts, so its specific installation location is generally the inner liner of the tire, which has a certain distance y from the neutral layer, and according to the analysis of a point of strain on the flexible ring in Section 2, the actual strain value can be approximated as

(58) $$\epsilon_{\theta }=\frac{y}{R^{2}}\left(\frac{\partial v}{\partial \theta }-\frac{\partial^{2}w}{\partial \theta^{2}}\right)$$

So according to the expression for displacement w, which is v in the previous section, the value of strain at the inner liner can be obtained as follows:

(59) $$\begin{array}{c} \varepsilon_{\theta }(\phi )=\overset{\infty }{\underset{n=1}{\sum }}\frac{y}{R^{2}}(n-n^{3})[(\alpha_{n3}A_{n2}+\alpha_{n1}A_{n1})cos\;(n(\phi )-\gamma_{n}))-(\alpha_{n2}A_{n1}-\alpha_{n4}A_{n2})sin\;(n(\phi )-\gamma_{n}))] \end{array}$$

The derived strain equation not only describes the mechanical response due to wear but also guides the application of uniaxial strain gauges in intelligent tires. Although this model is based on strain research, it also requires the participation of other sensors. The model is constructed with the initial tire pressure considered, so the tire pressure sensor is needed to work with the role as part of the model’s inputs. In addition, the collection of tire loads in the real application also requires the integration of force sensors in the car chassis for cooperation. Therefore, the model is always inextricably linked to the application of the sensors.

## 4. Model Validation

### 4.1. Parameter Acquisition

According to the analysis of the number of modal superpositions in Gong [37], the displacement amplitude of the signal is almost unaffected when the modal superposition is taken to be 30 or more, but to allow the calculated curves to be as smooth as possible while retaining the features, the value of n is taken to be 60 in the subsequent work.

**Figure 7:**
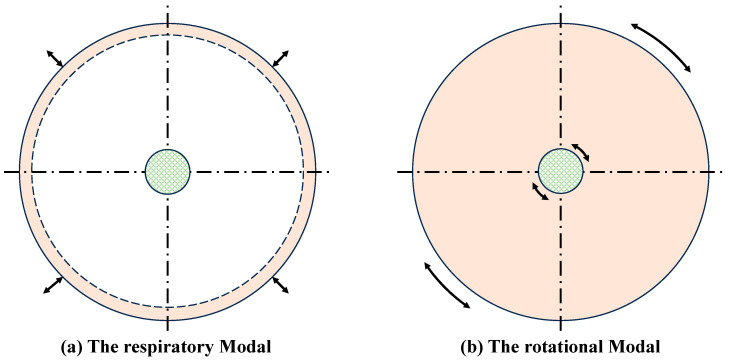
Schematic diagram of the zero-order mode of free tire vibration. (**a**) represents the respiratory modal in the radial direction, and (**b**) represents the rotational modal in the circumferential direction. The dash line represents the starting position of the ring, the solid line represents the vibration shape under this mode, and the arrow is used to indicate the vibration direction.

The numerical simulation accuracy of the tire physical model directly depends on the accuracy of the input parameters. To ensure the mechanical equivalence between the model and the actual tire, it is necessary to obtain the relevant geometric parameters and material physical parameters. The geometric parameters can be obtained by measuring the two-dimensional cross-section drawings of the tire and the finite element modeling process. The physical parameters of the material can be obtained by the natural frequency of the tire, and the natural frequency can be obtained by finite element modal analysis or experiments. According to the analysis of the tire modal by Gong [37] and Wei et al. [47,48]. It is known that free vibration differential equations can be obtained by derivation from the control equations of the model. According to the analysis of the literature, the intrinsic frequency of the zeroth-order modal under the free vibration of non-rotating tires can be solved at n=0. There are a total of four solutions, among which the two non-zero solutions are $\omega_{01}$ and $\omega_{02}$, which correspond to the respiratory modal (radial outward extension) and the rotational modal (circumferential rotation), as shown in Figure 7. and the higher frequency value corresponds to the breathing mode. The radial and tangential stiffness parameters of the tire can be obtained by substituting the 0th-order intrinsic frequency values obtained from the simulation into Equation (60). The parameters obtained are shown in Table 1.

(60) $$\begin{array}{l} \omega_{01}=\sqrt{\frac{1}{\rho A}\left(\frac{EA}{R^{2}}-\frac{p_{0}b}{R}+k_{w}\right)}\\ \omega_{02}=\sqrt{\frac{I_{r}+2\pi \rho AR^{3}}{I_{r}\rho A}k_{v}} \end{array}$$

### 4.2. Finite Element Simulation Verification

Due to the temporary lack of test conditions, the construction of the intelligent tire system is more complex, the period is longer, and the improved model is still in the theoretical research stage. Therefore, the finite element model is used for preliminary verification.

**Figure 8:**
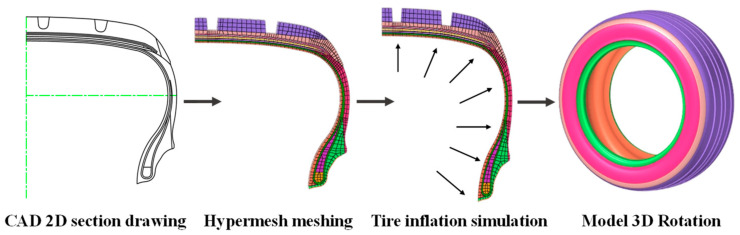
The modeling process of finite element model. The different colored parts in the finite element model represent different structures in the tire, such as tread, belt layer, inner liner, etc.

**Figure 9:**
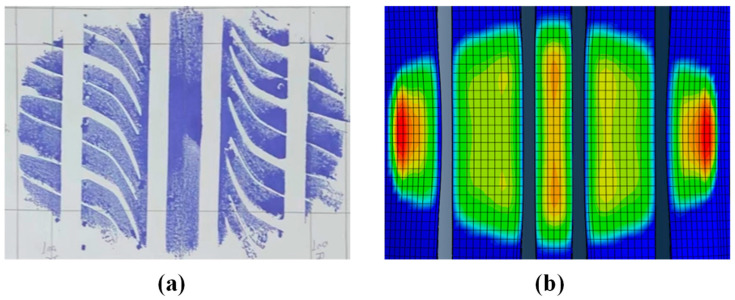
Contact pressure imprint test: (**a**) test results; (**b**) finite element simulation results, and the different color areas in the finite element simulation results represent the stress distribution in the grounding area.

**Figure 10:**
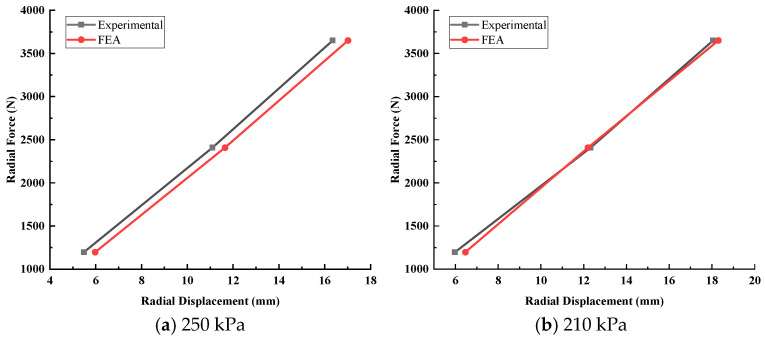
Stiffness test at different tire pressures. (**a**) shows the stiffness verification at a tire pressure of 250 kPa. (**b**) shows the stiffness verification at a tire pressure of 210 kPa.

The main process of establishing the finite element model of the tire is as follows: The two-dimensional section diagram of the tire carcass is geometrically simplified by AutoCAD2022 and then imported into HyperMesh2019 to establish a two-dimensional mesh model. The ABAQUS2019 software is used to simulate the inflation, so that the corresponding tire pressure value is reached in the tire; the three-dimensional solid mesh is generated by *SYMMETRIC MODEL GENERATION, REVOLVE; and then the model is loaded and constrained to complete the free-rolling simulation of the tire under load, as shown in Figure 8. The tire is a 205/55R16 radial tire, and the model has no complex pattern. In our previous study [49], the validity of the finite element model was verified by the tire contact pressure imprint test (Figure 9), and the stiffness test under different tire pressures (Figure 10).

The purpose of the model improvement is to integrate the use state of the tire into the physical model and use the circumferential strain signal calculated by the model to develop the related algorithm of the intelligent tire. Therefore, if the finite element model can be used to verify the accuracy of the physical model, it shows that the model has application value. Under the premise of keeping other parameters consistent, the tire pressure and load are set as simulation variables by the control variable method: the tire pressure change range is set to 190–230 kPa (190, 210, 230 kPa three gradients), and the load change range covers 3000–4600 N (five loading levels are set at 400 N intervals).

**Figure 11:**
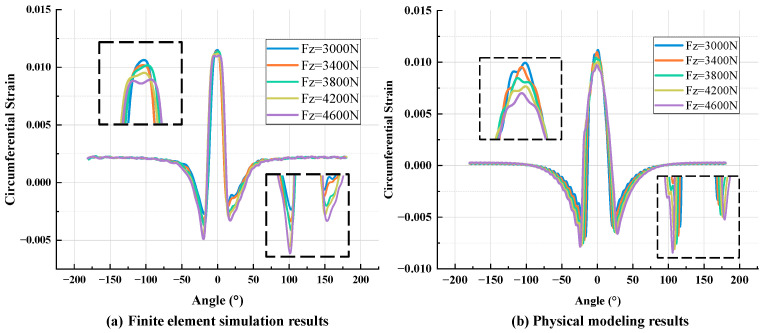
Strain signal curves under different loads: (**a**) finite element simulation; (**b**) physical model calculation.

**Figure 12:**
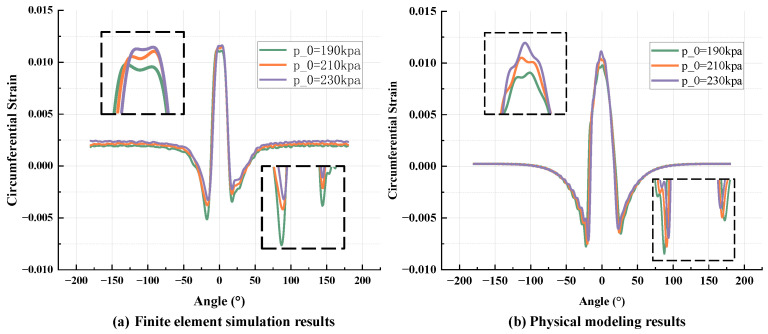
Strain signal curves under different tire pressures: (**a**) finite element simulation; (**b**) physical model calculation.

Figure 11 shows the strain signal values extracted from the tire inner liner under different loads when the tire pressure is 210 kpa and the speed is 40 km/h. Figure 12 shows the strain signal values extracted from the tire inner liner under different tire pressures when the load is 3400 N and the speed is 40 km/h. The comparison results of Figure 11a,b show that the overall change trend of the two is always consistent. At the peak of the tensile strain, both of them decrease with the increase in the load, and at the two peaks of the compressive strain, both of them increase with the increase in the load. The results calculated by the physical model show asymmetry at the peak of compressive strain, which is due to the consideration of the tire-damping effect in the model. In the finite element simulation, the tire adopts the Neo-Hookean constitutive model, which does not directly include damping-related parameters, so there is no obvious asymmetry. For Figure 12a,b, the trend of the two curves also maintains a high degree of consistency. With the increase in tire pressure, the peak value of tensile strain increases and the peak value of compressive strain decreases. Asymmetry can also be explained in the same way as above.

According to the above comparison and verification, it can be seen that the results calculated by the physical model can reflect the trend of the strain signal under different variables well, indicating that the characteristics of the strain signal reflected by the improved model are reliable and meaningful.

## 5. Exploring the Effect of Wear on Tire Strain

The core difference between this study and the existing literature research is that the dynamic wear evolution characteristics in the actual use of tires are incorporated into the construction of the tire multi-physical field coupling model. Because tread wear is a key parameter to characterize the degradation of tire life and mechanical properties, the introduction of this variable significantly improves the engineering applicability of the model. As described in Section 4, the numerical simulation based on Abaqus2019 has effectively verified the reliability of the improved model. To further reveal the influence mechanism of wear on the mechanical response of tires, this section continues to use the method of combining the finite element model and physical model calculations to systematically compare and analyze the evolution of the circumferential strain signals of tires under different wear degrees (0–6 mm). The simulation data are obtained from the linear rolling condition under specific working condition parameters (tire pressure 210 kPa, vertical load 3800 N, rolling speed 40 km/h).

**Figure 13:**
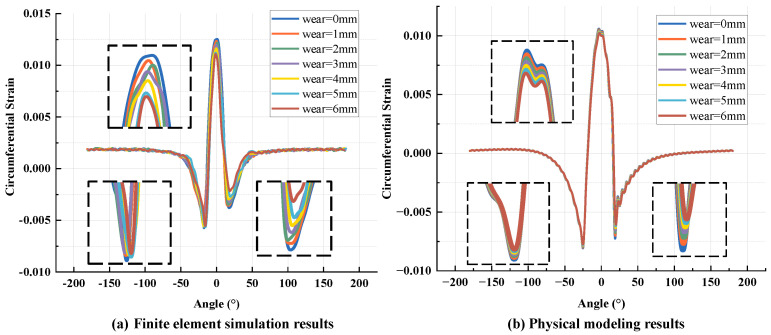
Strain signal curves under different wear: (**a**) finite element simulation; (**b**) physical model calculation.

Figure 13a shows the strain signal plots at different wear amounts, and it can be observed that the tensile–compressive peaks of the strain signal all decrease with the increase in the wear amount after local magnification processing. This is because, in the structural distribution of tires, the stiffness of various belts composed of steel cords is much greater than that of rubber. Therefore, it is considered that the neutral axis position of the tire cross-section remains unchanged before and after wear. However, when the tire tread wears, the tread becomes thinner, the moment arm becomes smaller, and the bending moment generated by the tangential force becomes smaller, so the tire deformation decreases, that is, the tensile compression peak of the strain decreases.

Figure 13b is the strain signal generated by the improved physical model, and the trend is consistent with the finite element model. In addition, based on the two plots, it can be seen that the asymmetry of the strain signal changes as the amount of wear increases. In the research results of K. Nishiyama [50], it is mentioned that the influence of tire wear on the strain signal is mainly reflected in the trailing edge strain, and the difference between the trailing edge strain and the leading edge strain is increasing, that is, the change in the second compressive strain peak in the signal increases with the increase in wear. This is because the tire tread is arc-shaped, and the radius of the shoulder is different from that of the center of the tire. However, when the tire is rolling, the shoulder and the center of the tire are simultaneously deformed when the measuring point enters the contact area after loading, and the recovery deformation of the shoulder is earlier than that of the center of the tire when leaving the contact area, which causes the shear deformation of the longitudinal force on the center of the tire to become larger, and this deformation produces tensile strain, so the overall compressive strain value at the trailing edge position becomes smaller. When the tire appears to be worn, the overall volume of the tread becomes less and the shear stiffness decreases, which results in a larger shear strain, so the overall compressive strain at the trailing edge position should be smaller. The strain signal calculated by the physical model established in this paper also has this feature, indicating that the influence of wear on the strain signal can be well reflected.

By analyzing the influence of wear on the strain signal, these characteristics or trends can be used in the next stage of research to reasonably judge or predict the wear of the tire during driving, which is of great value for future research. Meanwhile, in practical sensor applications, the correlation characteristics between the wear and strain signals can ensure that the raw sensor signals can be directly fed into downstream algorithms without complex pre-processing.

## 6. Summary and Perspectives

In this paper, the traditional flexible ring model is improved to incorporate tire wear into the dynamic behavior of tires within the theoretical framework. In this, the tread is simplified as a brush, and the mechanical equilibrium equations of the tread unit are established. By introducing the pressure distribution of the tire and combining the equations of physics and motion, the quantitative expression of the strain is finally obtained. To verify the reliability of the physical model, this study used finite element simulation to systematically validate the improved model. By comparing the numerical trends of the strain signals under different loads and inflation pressures, it is confirmed that the model calculation results are in good agreement with the numerical simulation results. Notably, the present model successfully reveals the relationship between tire wear and strain signals: the degree of tire wear has a negative correlation with the peak tensile and compressive strains. This provides a theoretical basis for the inversion of tire wear state based on strain signals, which is of great engineering value for the condition monitoring of intelligent tires and the optimization of motion control algorithms for autonomous driving systems.

This study improves an existing model which can generate circumferential strain signals for tires. This signal acquisition method can be used as an alternative to experimental acquisition and finite element simulation extraction, thus allowing the construction of a baseline dataset for tire condition estimation. While the experimental validation of sensor integration is beyond the current scope for us, the model’s ability to generate strain datasets lays the foundation for a data-driven intelligent tire system. These datasets are critical for training machine learning models to estimate tire–road interaction parameters from sparse sensor measurements. The use of physical models can effectively reduce the cost of experiments or finite element simulations and significantly improve the efficiency of intelligent tire system research and development. In the future, by integrating artificial intelligence algorithms and big data analysis techniques, this model can be further developed into a digital twin platform for tire state prediction over an entire life cycle, and this opens up a new direction for research on the integration of tire mechanical modeling and intelligent tire perception technologies.

This paper improves a new theoretical model of intelligent tires to take wear into account while showing the effect of wear on the strain signal. However, a more comprehensive model needs to consider the influences of strain signals in more dimensions, such as taking temperature into account. The temperature has a significant effect on the rubber’s intrinsic behavior, tire pressure dynamics, and friction heat generation mechanism, which may require further in-depth research. Meanwhile, the model can be extended to study three dimensions to take the composite slip in the brush model into account and to carry out the transverse mechanics study of tires. In addition, after the model has been refined and validated to a certain extent, more intelligent algorithms can be utilized to estimate the state parameters of the tire, and bench tests as well as real-vehicle tests can be constructed for further validation of the model.

## Figures and Tables

**Table 1:** The tire-related parameters required for the model.

Parameter Name	Symbols	Value
Thickness of ring	h (m)	0.006
Thickness of tread	l (m)	0.01076
Distance to the inner liner	y (m)	0.00315
Radius of the ring	R (m)	0.3023
Width of cross-section	b (m)	0.189
Area of cross-section	A (m^2^)	b × h
Radial stiffness	kw (N/m)	9.5 × 10^5^
Tangential stiffness	kv (N/m)	2.1 × 10^5^

## Data Availability

Data are contained within the article.
